# DDR2 facilitates hepatocellular carcinoma invasion and metastasis via activating ERK signaling and stabilizing SNAIL1

**DOI:** 10.1186/s13046-015-0218-6

**Published:** 2015-09-11

**Authors:** Binhui Xie, Weihao Lin, Junming Ye, Xiaonong Wang, Bing Zhang, Shiqiu Xiong, Heping Li, Guosheng Tan

**Affiliations:** Department of Hepatobiliary Surgery, The First Affiliated Hospital of Gannan Medical University, Ganzhou, 341000 China; Department of General Surgery, The First Affiliated Hospital of Sun Yat-sen University, Guangzhou, 510080 China; Department of Anesthesiology, The First Affiliated Hospital of Gannan Medical University, Ganzhou, 341000 China; Department of Medical Imaging, The First Affiliated Hospital of Sun Yat-sen University, Guangzhou, 510080 China; Department of Biochemistry, University of Leicester, Leicester, LE1 9HN UK

**Keywords:** DDR2, HCC, ERK, SNAIL1, EMT

## Abstract

**Background:**

Several studies have found that DDR2 is up-regulated in many tumor types and facilitates tumor progression. However, the role of DDR2 in hepatocellular carcinoma (HCC) progression and its downstream signaling pathways remain unclear.

**Methods:**

DDR2 expression was assessed in several cell lines and 112 pairs of HCC and matched adjacent noncancerous liver tissues. Clinical significance of DDR2 in HCC was analyzed. Phosphorylated DDR2 (p-DDR2) expression was detected by immunoblotting to evaluate its correlation with DDR2. The effect of DDR2 on HCC cell migration and invasion were examined. Cycloheximide chase experiments were performed to detect the half-life of SNAIL1. Moreover, DDR2 expression was detected by immunohistochemistry to evaluate its correlation with SNAIL1. The regulatory effect of DDR2 on ERK signaling, SNAIL1, EMT, MT1-MMP and MMP2 was confirmed by immunoblotting. The effect of type I collagen on DDR2/ERK2/SNAIL1 signaling was assessed.

**Results:**

DDR2 was more highly expressed in HCC than in non-HCC tissues. DDR2 overexpression was correlated with clinicopathological features of poor prognosis. Clinical analysis revealed that DDR2 is an independent prognostic marker for predicting overall survival and disease free survival of HCC patients. Overexpression of DDR2 is associated with p-DDR2 amplification. In vitro studies showed that DDR2 facilitates HCC cell invasion, migration and EMT via activating ERK2 and stabilizing SNAIL1. DDR2 can up-regulate MT1-MMP and MMP2 expression through ERK2/SNAIL1 signaling in HCC. Additionally, collagen I can induce DDR2/ERK2/SNAIL1 signaling activation in HCC cells.

**Conclusions:**

Our findings suggest that DDR2 plays an important role in promoting HCC cell invasion and migration, and may serve as a novel therapeutic target in HCC.

## Background

Hepatocellular carcinoma (HCC) is one of the most prevalent malignant tumors worldwide and is the second leading cause of cancer-related death in China [1–3]. In recent years, the incidence of HCC is increasing in many parts of the world including the United States, partly due to the rise in hepatitis C virus infection [4–6]. Hepatic resection offers the chance of a cure for patients with HCC, but the prognosis remains very poor [7, 8]. Hence, understanding the molecular mechanisms involved in hepatocarcinogenesis and exploring prognostic markers and therapeutic targets of HCC is urgently needed.

The discoidin domain receptors (DDRs), consisting of DDR1 and DDR2, are distinctive receptor tyrosine kinases (RTKs) and signal in response to collagens instead of soluble peptide growth factors [9–11]. Multiple studies have suggested that DDR2 can regulate cell adhesion, migration and matrix remodeling [12, 13]. DDR2 have also been shown to exhibit aberrant expression patterns in several tumor types, including nasopharyngeal and prostate cancer [14–16]. Furthermore, it was found that the abnormal expression of DDR2 implicated in cancer progression and a poor prognosis [17, 18]. However, the role of DDR2 in HCC and the molecular mechanisms by which DDR2 exerts its biological function are still unclear.

SNAIL1, a member of transcription factors, is critical for inducing and sustaining cancer epithelial–mesenchymal transition (EMT) [19, 20]. SNAIL1-induced EMT has been demonstrated essential for promoting tumor cell migration and invasion in multiple tumor types [21, 22]. Recently, Zhang *et al*. have found that SNAIL1 is regulated by the DDR2/ERK2 signaling pathway in breast cancer [23]. In the previous studies, ERK signaling pathway has been considered to promote the progression of HCC [24, 25]. It is also known that SNAIL1 plays an essential role in regulating HCC EMT [26, 27]. However, whether DDR2 is implicated in regulating the ERK signaling and SNAIL1 in HCC has not been clarified.

As representative epithelial-specific and mesenchymal-specific biomarkers, E-cadherin and Vimentin are crucial factors in the invasion, migration and EMT of HCC [21]. Down-regulation of E-cadherin and up-regulation of Vimentin are used as biomarkers indicating an epithelial cell has undergone EMT in HCC [21]. Multiple studies have revealed that EMT associated with patients poor prognosis is an important step in the invasion and migration of HCC [21, 26]. Importantly, SNAIL1-induced E-cadherin down-regulation and Vimentin up-regulation have been demonstrated essential for triggering the EMT of HCC [27]. However, whether DDR2 is implicated in regulating the expression of E-cadherin and Vimentin in HCC are still unknown.

In this study, we found that DDR2 was more highly expressed in HCC tissues than that in non-tumor tissues, and DDR2 overexpression was correlated with poor clinicopathological features and outcome of HCC patients. Our study demonstrated that DDR2 has an oncogenic role in HCC tumorigenesis by facilitating cancer cell invasion, migration and epithelial–mesenchymal transition via activating ERK signaling and stabilizing SNAIL1. Additionally, DDR2 can up-regulate membrane type-1 matrix metalloproteinase (MT1-MMP) and MMP2 expression through ERK2/SNAIL1 signaling in HCC. Our study identified that DDR2 is a novel regulator of EMT through stabilizing SNAIL1, indicating its potential therapeutic value for reducing HCC invasion and metastasis.

## Materials and methods

### Tissue samples

A total of 112 pairs of HCC and corresponding adjacent non-tumorous liver tissues (>2.0 cm from the resection margin) were obtained from patients undergoing hepatectomy between February 2007 and December 2009 at the Department of Hepatobiliary Surgery, the First Affiliated Hospital of Gannan Medical University. The patient characteristics are shown in Table 1. All patients provided written informed consent before hepatectomy, and our study were approved by the Ethics Committee of the First Affiliated Hospital of Gannan Medical University according to the 1975 Declaration of Helsinki.

**Table 1:** Correlations between DDR2 expression and clinicopathologic features in HCC

Characteristics	n	DDR2 expression		*P* value
		High	Low	
Gender				
Female	25	15	10	
Male	87	41	46	0.364
Age (year)				
≤ 45	29	10	19	
> 45	83	46	37	0.083
HBsAg status^a^				
Negative	22	7	15	
Positive	90	49	41	0.095
Cirrhosis				
No	37	15	22	
Yes	75	41	34	0.228
AFP (μg/l)^b^				
≤ 400	55	23	32	
> 400	57	33	24	0.130
Tumor size				
≤ 5 cm	37	14	23	
> 5 cm	75	42	33	0.107
Tumor number				
Single	86	37	49	
Multiple	26	19	7	0.013^*^
Tumor capsule				
Complete	20	7	13	
Incomplete	92	49	43	0.217
Vascular invasion				
No	86	36	50	
Yes	26	20	6	0.003^*^
Edmondson grade				
I/II	64	24	40	
III/IV	48	32	16	0.004^*^
TNM stage				
I/II	67	25	42	
III/IV	45	31	14	0.002^*^

^a^ *HbsAg* hepatitis B surface antigen, ^b^ *AFP* a-fetoprotein, *P < 0.05

### Cell lines

The immortalized normal human liver cell line L02 and five HCC cell lines including SMMC-7721, Huh-7, HepG2, Hep3B and MHCC-97H were obtained from from the Type Culture Collection of the Chinese Academy of Sciences (Shanghai, China). All cell lines were cultured in Dulbecco’s modified Eagle medium (DMEM, Gibco, Grand Island, NY, USA) containing 10 % fetal bovine serum (FBS, Gibco).

### Quantitative reverse transcription-polymerase chain reaction (qRT-PCR)

Total RNA was extracted using TRIzol reagent (Invitrogen, USA). qRT-PCR was done in an ABI 7500 system using the SYBR® Premix Ex Taq™ II (Tli RNaseH Plus) (TakaRa, Japan). DDR2 primers: forward 5’-CTCCCAGAATTTGCTCCAG-3’; reverse 5’-GCCACATCTTTTCCTGAGA-3-3’. SNAIL1 primers: forward 5’- GCT CCACAAGCACCAAGAGT-3’; reverse 5’- ATTCCATGGCAGTGAGAAGG-3’. β-actin primers: forward 5’-GGGAA ATCGTGCGTGACAT-3’; reverse 5’-C TGGA AGGTGGACAGCGAG-3’.

### Western immunoblotting

The following primary antibodies were used in the western immunoblotting assays: SNAIL1 (ab180714, Abcam, Cambridge, UK), Vimentin (ab137321, Abcam, Cambridge, UK), E-cadherin (ab15148, Abcam, Cambridge, UK), MT1-MMP (ab53712, Abcam, Cambridge, UK), DDR2 (sc-8989, Santa Cruz, CA, USA), p-DDR2 (MAB25382, R&D systems Inc, USA), ERK1/2 (sc-292838, Santa Cruz, CA, USA), p-ERK1/2 (sc-101760, Santa Cruz, CA, USA), MMP2 (sc-10736, Santa Cruz, CA, USA) and β-actin polyclonal antibody (sc-130656, Santa Cruz, CA, USA). Immunoblotting assays were performed as reported previously [3].

### Immunohistochemical staining

Immunohistochemical assay was performed on paraformaldehyde-fixed paraffin sections as previous reported [28]. The DDR2 (sc-8989, Santa Cruz, CA, USA) and SNAIL1 (ab180714, Abcam, Cambridge, UK) primary antibodies were used at a 1:100 dilution in the immunohistochemistry assays. The immunostaining intensity and average percentage of positive cells were evaluated as previous reported [28]. Immunostaining intensity was evaluated as four grades: 0, negative; 1, weak; 2, moderate; 3, strong. And the percentage of positive cells was categorized as the following grades: 0, 0 %; 1, 1 to 10 %; 2, 11 to 50 %; 3, 51 to 80 %; and 4 > 80 %. The immunostaining intensity and average percentage of positive cells were evaluated for ten independent high magnification fields. By multiplying the staining intensity and the percentage of positive cells, the final weighed expression score was obtained (0–12).

### Expressing plasmids and RNAi transfection

The DDR2 expressing plasmid and its empty plasmid pCMV6-XL6 were both obtained from Origene Technologies Inc. (Rockville, USA). DDR2 expressing plasmid or empty plasmid pCMV6-XL6 was transfected into Hep3B cells using TurboFectin Transfection Reagent purchased from Origene Technologies Inc. (Rockville, USA). The DDR2 shRNA and scrambled shRNA vector pRS were also purchased from Origene Technologies Inc. (Rockville, USA). DDR2 unique 29mer shRNA constructs in pRS Vector or scrambled shRNA vector pRS was transfected into MHCC-97H cells using TurboFectin Transfection Reagent purchased from Origene Technologies Inc. (Rockville, USA). The specific siRNA sequences against ERK2 (sc-35335), against SNAIL1 (sc-38398) and the scramble siRNAs (sc-37007) were obtained from Santa Cruz Biotechnology (Santa Cruz, USA).

### Cycloheximide (CHX) chase experiments

To assess SNAIL1 protein stability, HCC cells were treated with 50 mg/ml cycloheximide (final concentration, Sigma) following expressing plasmids or RNAi transfection for 72 h. At the given time points, HCC cells were harvested, flash-frozen, and analyzed by Western blotting as described above. SNAIL1 protein degradation rates were quantified by densitometry using time point zero as 100 %.

### Co-Immunoprecipitation assay

Total protein lysate was obtained in immunoprecipitation buffer. The lysate was precleared with protein A/G-agarose beads. Total protein in supernatants was qualified by BCA method. Then, total protein was diluted into 1 μg/μL with PBS and mixed with primary antibodies against DDR2, SNAIL1, ERK2 or IgG. The mixtures were shaken on rotating shaker for overnight at 4 °C. The supernatant was obtained and used in the immunoblotting assay.

### Transwell migration and invasion assays

Transwell migration assays were performed in 12 well plates with 8-μm BioCoat control inserts (Bedford, MA). HCC cells that were suspended in serum-free medium were added into the upper chamber and medium with 10 % FBS was added into the lower chamber. The cells on the inner layer were softly removed and the adherent tumor cells on undersurface of the insert were stained with crystal violet. Transwell invasion assays were performed in BioCoat Matrigel invasion chamber containing an 8 μm pore-size PET membrane with a uniform layer of BD Matrigel basement membrane matrix (Becton Dickinson Labware). The following protocols were the same as cell migration assays.

### Statistical analysis

Data were showed as the Mean ± SEM. Statistical significance was established, with the SPSS 16.0 statistical software package (SPSS Inc., Chicago, IL), using a two-tailed Student’s *t* test, a Kaplan–Meier plot, a log-rank test, a Spearman correlation coefficient analysis, a Chi-square test or Fisher’s exact test. Independent prognostic factors were assessed by the Cox proportional hazards stepwise regression model. P values were 2-sided and a *P* value of less than 0.05 was considered statistical significance.

## Results

### Up-regulation of DDR2 is a frequent event in HCC lines

**Fig. 1:**
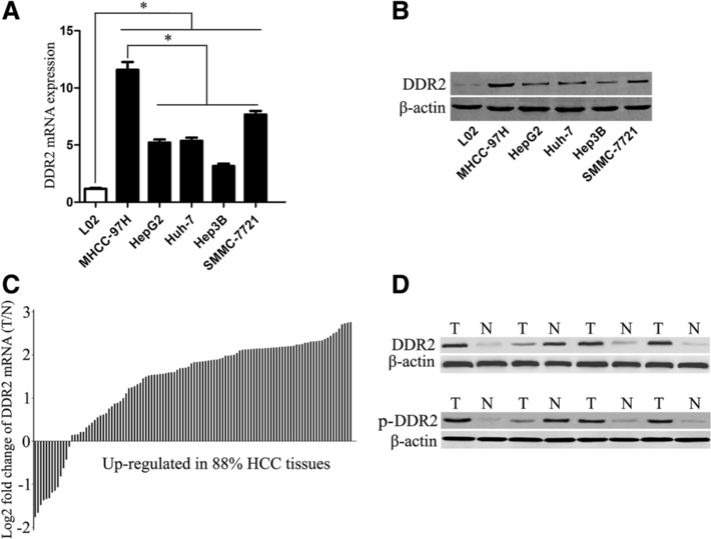
The expression of DDR2 in cell lines and HCC tissues. **a** DDR2 mRNA expression in the immortalized normal human liver cell line L-02 and five hepatoma cell lines (MHCC-97H, HepG2, Huh-7, Hep3B and SMMC-7721) using qRT-PCR (*n* = 3, ^*^ *P* < 0.01). **b** DDR2 protein expression in the immortalized normal human liver cell line L-02 and five hepatoma cell lines (MHCC-97H, HepG2, Huh-7, Hep3B and SMMC-7721) using immunoblotting (*n* = 3, *P* < 0.01). **c** DDR2 mRNA expression in HCC tissues (T) and corresponding adjacent no tumorous tissues (N) using qRT-PCR (*n* = 112, *P* < 0.01). **d** The protein expression of DDR2 and phosphorylated DDR2 (p-DDR2) in HCC tissues (T) and corresponding adjacent no tumorous tissues (N) using immunoblotting (*n* = 112, *P* < 0.01, respectively)

We first analyzed DDR2 expression in an immortalized non-tumourigenic hepatocyte cell line L02 and a panel of HCC cell lines (SMMC-7721, Huh-7, HepG2, Hep3B and MHCC-97H) using qRT-PCR and Western blotting. We found that DDR2 expression was evidently up-regulated in all HCC cell lines as compared with that in a non-tumourigenic hepatocyte cell line L02 (*P* < 0.01, respectively, Fig. 1a-b). Furthermore, DDR2 expression in the highly invasive HCC cell line (MHCC-97H) were evidently higher than those in the low invasive HCC cell lines including SMMC-7721, Huh-7, HepG2, Hep3B (*P* < 0.01, respectively, Fig. 1a-b). The characteristics of each cell line are shown in Table 2. Our data indicates that elevated DDR2 expression confers increased invasive potential of HCC cells. In our study, MHCC-97H expressed the highest expression level of DDR2 in HCC cell lines and the Hep3B expressed the lowest expression level of DDR2 among the five HCC cell lines. Thereby, Hep3B cell line was selected for DDR2 overexpression experiment here, while MHCC-97H cell line were used in DDDR2 knockdown experiment.

**Table 2:** Characteristics of the cell lines used in this study

Cell line	Type	Characteristics			
		HBsAg^a^	P53	AFP^b^	Invasion & migration
L02	Liver cell line	Negative	Wild type p53	Negative	No
Hep3B	Hepatoma cell line	Positive	P53 deletion	Positive	Low
HepG2	Hepatoma cell line	Negative	Wild type p53	Positive	Low
Huh-7	Hepatoma cell line	Negative	P53 mutation	Positive	Low
SMMC-7721	Hepatoma cell line	Negative	P53 mutation	Positive	Low
MHCC-97H	Hepatoma cell line	Positive	P53 mutation	Positive	High

^a^ *HbsAg* hepatitis B surface antigen, ^b^ *AFP* a-fetoprotein

### Clinical significance of DDR2 expression in HCC tissues

To analyze the clinical significance of DDR2 in HCC, we detected the DDR2 expression in 112 pairs of HCC and matched tumor-adjacent samples using qRT-PCR and Western blotting, and found that DDR2 expression was prominently higher in the HCC samples than that in the noncancerous samples (*P* < 0.01, respectively, Fig. 1c-d). In our study, we also detected the phosphorylated DDR2 (p-DDR2) expression in HCC, which was previously shown to be associated with DDR2 activation. We found that p-DDR2 expression level was evidently higher in the HCC samples than that in the noncancerous samples (*P* < 0.01, Fig. 1d). To further identify the clinical role of DDR2 in HCC, we detected the correlations of the DDR2 protein expression with clinicopathological characteristics, including gender, age, HBsAg status, AFP level, tumor size, liver cirrhosis, capsule formation, Edmondson-Steiner grade, tumor number, vascular invasion and TNM stage. In this study, the median DDR2 protein expression level was used as the cutoff point to divide into low-expressing and high expressing groups. Strikingly, DDR2 expression was evidently correlated with tumor number, vascular invasion, Edmondson-Steiner grade and TNM stage (Table 1). However, no relationship was found between the DDR2 expression and other clinicopathological characteristics including gender, age, HBsAg status, AFP level, tumor size, liver cirrhosis, capsule formation (Table 1).

### DDR2 expression is an independent prognostic factor for HCC

**Fig. 2:**
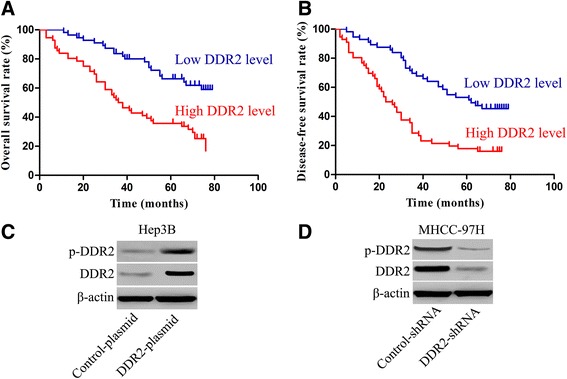
Prognostic value of DDR2 protein expression in HCC and its correlation with phosphorylated DDR2 (p-DDR2) in HCC cells. **a** Overall survival for DDR2 protein expression (log-rank, *P* < 0.01). **b** Disease-free survival for DDR2 protein expression (log-rank, *P* < 0.01). **c** Immunoblotting analysis of DDR2 and p-DDR2 in Hep3B cells with DDR2 expressing plasmid transfection or control plasmid transfection. **d** Immunoblotting analysis of DDR2 and p-DDR2 in MHCC-97H cells with DDR2-shRNA transfection or control-shRNA transfection

In this study, the median DDR2 protein expression was used as the cutoff point to divide into high-expressing and low-expressing groups for HCC patients’ survival. HCC patients in the high DDR2-expressing group had obviously reduced overall survival and disease-free survival. The 5-year overall survival rate of the high DDR2-expressing group was significantly lower than that of the low DDR2-expressing group (*P* < 0.01) (Fig. 2a). The 5-year disease-free survival rate of the high DDR2- expressing group was obviously lower than that of the low DDR2-expressing group (*P* < 0.01) (Fig. 2b). In our study, we also performed Univariate analysis and Cox proportional hazards regression analysis. The univariate analysis revealed that DDR2 level, HBsAg status, tumor number, vascular invasion, Edmondson-Steiner grade and TNM stage were the prognosis factors for HCC patients (Table 3). In a multivariate analysis model, DDR2 expression level was obviously associated with overall survival (HR 2.169; 95 % CI, 1.198–3.927; *P* < 0.05) and disease-free survival (HR 2.051; 95 % CI, 1.228–3.425; *P* < 0.05) (Table 4). These data indicates that the DDR2 expression level is an independent prognosis factors of HCC patients.

**Table 3:** Univariate prognostic analysis of overall survival and disease-free survival in HCC patients

Variable	n	Overall survival rate (%)			Disease-free survival rate (%)		
		3y	5y	*P* value	3y	5y	*P* value
Gender							
Female	25	87.7	66.8		56.0	44.0	
Male	87	60.9	46.4	0.100	47.1	31.8	0.234
Age (year)							
≤ 45	29	82.4	57.3		62.1	37.9	
> 45	83	61.4	40.8	0.417	44.6	34.7	0.639
HBsAg status^a^							
Negative	22	90.5	85.4		72.7	59.1	
Positive	90	61.1	41.4	0.008^*^	43.3	29.6	0.017^*^
Cirrhosis							
No	37	64.2	58.1		51.4	39.9	
Yes	75	68.0	47.7	0.440	48.0	33.3	0.457
AFP (μg/l)^b^							
≤ 400	55	61.3	53.4		49.1	39.7	
> 400	57	71.9	48.7	0.699	49.1	31.6	0.714
Tumor size							
≤ 5 cm	37	75.7	58.9		51.4	39.9	
> 5 cm	75	62.3	46.8	0.398	48.0	33.3	0.938
Tumor number							
Single	86	71.8	57.1		55.8	43.9	
Multiple	26	50.0	30.8	<0.001^*^	26.9	7.7	<0.001^*^
Tumor capsule							
Complete	20	75.0	52.2		50.0	44.4	
Incomplete	92	65.0	50.5	0.507	48.9	33.7	0.611
Vascular invasion							
No	86	67.2	55.0		53.5	41.6	
Yes	26	65.4	37.1	0.011^*^	34.6	15.4	0.004^*^
Edmondson grade							
I/II	64	76.3	56.0		64.1	44.8	
III/IV	48	54.2	43.7	0.027^*^	29.2	22.9	0.002^*^
TNM stage							
I/II	67	69.8	62.0		58.2	47.5	
III/IV	45	62.2	34.0	<0.001^*^	35.6	17.8	0.001^*^
DDR2 level							
Low	56	83.7	66.3		69.6	53.1	
High	56	50.0	35.7	<0.001^*^	28.6	17.9	<0.001^*^

^a^ *HbsAg* hepatitis B surface antigen, ^b^ *AFP* a-fetoprotein, *P < 0.05

**Table 4:** Multivariate analysis of factors contributing to overall survival and disease-free survival in HCC patients

Variable	Overall survival rate		Disease-free survival rate	
	HR (95 % CI)^a^	*P* value	HR (95 % CI)^a^	*P* value
HBsAg status^b^	3.021(1.335–6.836)	0.008^*^	2.556 (1.300–5.026)	0.007^*^
Tumor number	2.374 (1.117–5.045)	0.025^*^	2.758 (1.363–5.579)	0.005^*^
Vascular invasion	0.912 (0.451–1.847)	0.799	1.086 (0.553–2.136)	0.810
TNM stage	1.269 (0.579–2.784)	0.552	0.950 (0.463–1.953)	0.890
Edmondson grade	0.999 (0.574–1.739)	0.998	1.389 (0.854–2.260)	0.185
DDR2 level	2.169 (1.198–3.927)	0.011^*^	2.051 (1.228–3.425)	0.006^*^

^a^ *HR* hazard ratio, ^b^ *HbsAg* hepatitis B surface antigen, *CI* confidence interval, *P < 0.05

### Overexpression of DDR2 is associated with DDR2 activation

To explore whether DDR2 overexpression is associated with DDR2 activation, we detected the phosphorylated DDR2 (p-DDR2) expression in HCC cells, which was previously shown to be associated with DDR2 activation in several studies. As expected, overexpression of DDR2 evidently increased the level of p-DDR2 in Hep3B cells (Fig. 2c). Furthermore, knockdown of DDR2 expression markedly attenuated the phosphorylation of DDR2 in MHCC-97H cells (Fig. 2d). Our results indicate that DDR2 amplification is associated with DDR2 activation.

### DDR2 promotes EMT and enhances invasion and migration ability in HCC

**Fig. 3:**
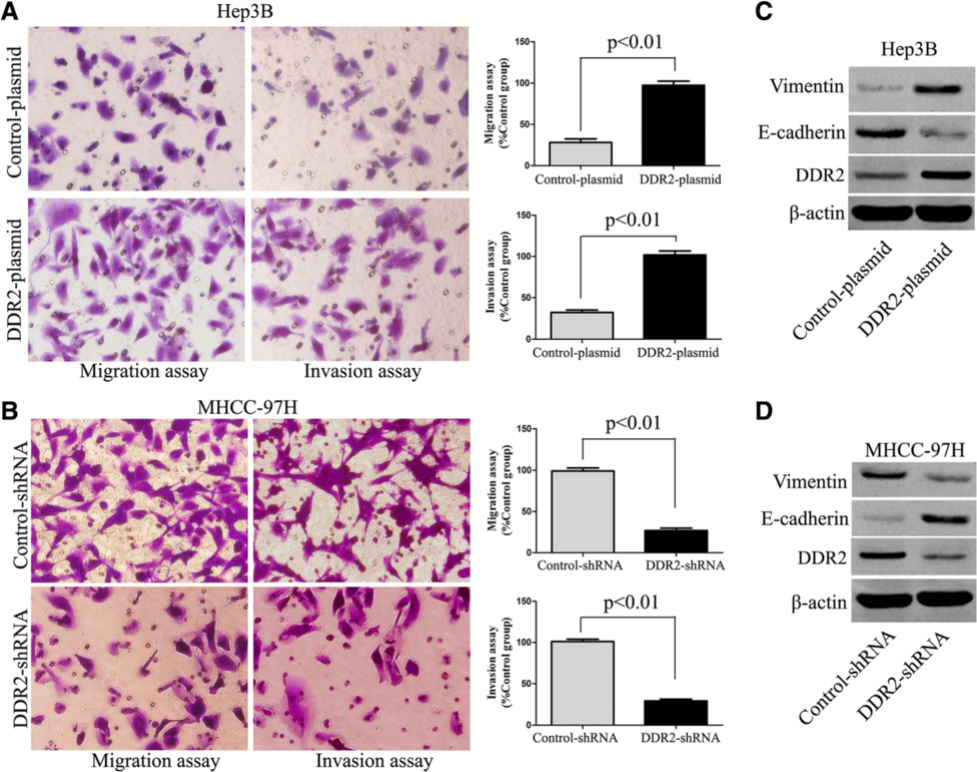
DDR regulates HCC cell mobility and EMT phenotype. **a** Cell migration and invasion as measured by Transwell assays were promoted by up-regulation of DDR2 in Hep3B cells as compared with control cells (*n* = 6, *P* < 0.01). **b** Cell migration and invasion as measured by Transwell assays were inhibited by down-regulation of DDR2 in MHCC-97H cells as compared with control cells (*n* = 6, *P* < 0.01). **c** Immunoblotting analysis of E-cadherin and Vimentin in Hep3B cells with DDR2 expressing plasmid transfection or control plasmid transfection (*n* = 6, *P* < 0.01). **d** Immunoblotting analysis of E-cadherin and Vimentin in MHCC-97H cells with DDR2-shRNA transfection or control-shRNA transfection (*n* = 6, *P* < 0.01)

We up-regulated DDR2 expression in Hep3B cells by DDR2 expressing plasmid. Transwell assays were performed to detect the effect of altering DDR2 expression levels on HCC cell invasion and migration. In our study, we found that up-regulation of DDR2 expression obviously promoted HCC cell migration and invasion (*P* < 0.01, respectively, Fig. 3a). Next, we down-regulated DDR2 expression by DDR2-shRNA vectors in MHCC-97H cells. As expected, silencing DDR2 was found to inhibit MHCC-97H cell migration and invasion by Transwell assays (*P* < 0.05, respectively, Fig. 3b). Moreover, DDR2 overexpression-induced EMT was associated with reduced E-cadherin expression and elevated Vimentin expression in Hep3B cells (*P* < 0.01, respectively, Fig. 3c). Otherwise, silencing DDR2-induced suppression of EMT in MHCC-97H cells was associated with elevated E-cadherin expression and reduced Vimentin expression. Our data indicate that DDR2 expression is associated with EMT phenotype and tumor cell migration and invasion in HCC (*P* < 0.01, respectively, Fig. 3d).

### DDR2 facilitates EMT via activating ERK signaling and stabilizing SNAIL1

**Fig. 4:**
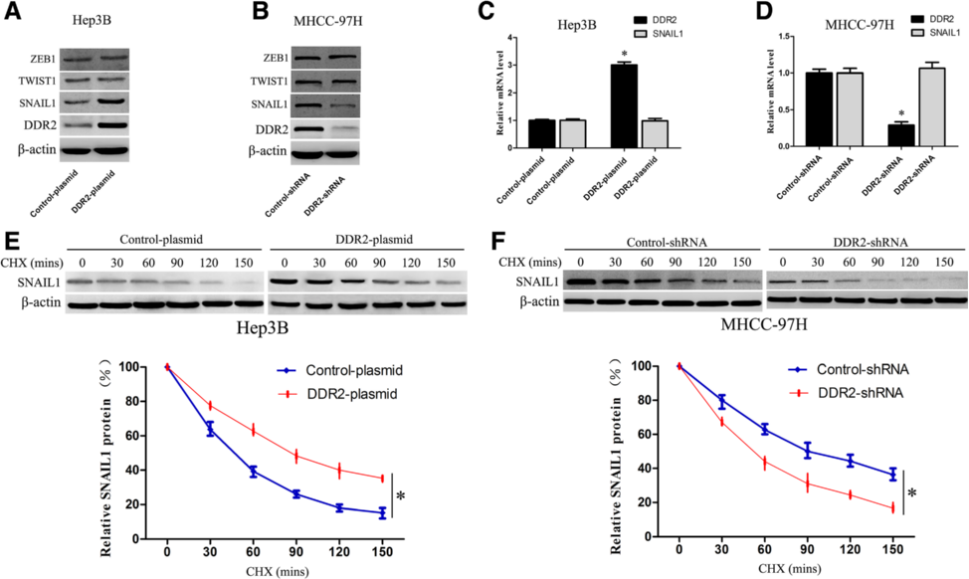
The effect of DDR2 on the expression of SNAIL1, TWIST1 and ZEB1 in HCC cells. **a** Immunoblotting analysis of SNAIL1, TWIST1 and ZEB1 in Hep3B cells with DDR2 expressing plasmid transfection or control plasmid transfection (*n* = 3, *P* < 0.01). **b** Immunoblotting analysis of SNAIL1, TWIST1 and ZEB1 in MHCC-97H cells with DDR2-shRNA transfection or control-shRNA transfection (*n* = 3, *P* < 0.01). **c** qRT-PCR analysis of SNAIL1 in Hep3B cells with DDR2 expressing plasmid transfection or control plasmid transfection (*n* = 3, *P < 0.01). **d** qRT-PCR analysis of SNAIL1 in MHCC-97H cells with DDR2-shRNA transfection or control-shRNA transfection (*n* = 3, *P < 0.01). **e** Cycloheximide (CHX) treated samples analysis following DDR2 expressing plasmid transfection or control plasmid transfection in Hep3B cells (*n* = 3, *P < 0.01). **f** Cycloheximide (CHX) treated samples analysis following DDR2-shRNA transfection or control-shRNA transfection in MHCC-97H cells (*n* = 3, *P < 0.01)

To explore the underlying mechanism by which DDR2 regulates cell migration, invasion and EMT in HCC, we detected the effect of DDR2 on several transcription factors considered to be inducers of EMT, such as SNAIL1, TWIST1 and ZEB1. We found that up-regulation of DDR2 in Hep3B cells increased the SNAIL1 protein expression and knockdown of DDR2 in MHCC-97H cells resulted in a decreased level of SNAIL1 protein. However, overexpression or depletion of DDR2 did not alter the expression of TWIST1 and ZEB1. (Fig. 4a-b). Notably, overexpression or depletion of DDR2 did not alter SNAIL1 mRNA expression (Fig. 4c-d). Considering the fact that DDR2 controls the SNAIL1 expression posttranslationally, cycloheximide chase experiments were performed to detect the half-life of SNAIL1. As expected, DDR2 overexpression in Hep3B cells obviously increased the half-life of SNAIL1 and DDR2 depletion in MHCC-97H cells markedly decreased the half-life of SNAIL1 (Fig. 4e-f).

**Fig. 5:**
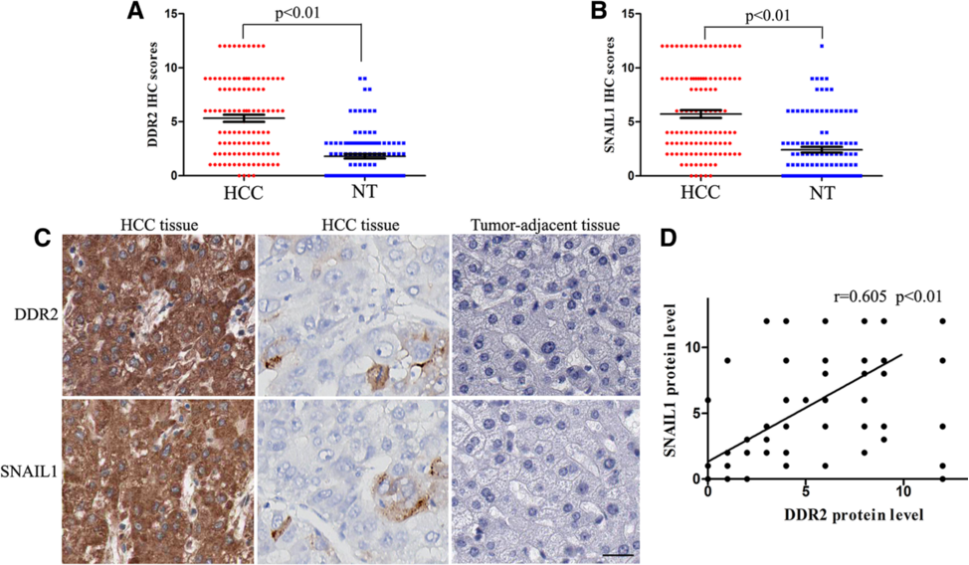
Immunohistochemical staining of DDR2 and SNAIL1 in HCC tissues. **a** The IHC scores of DDR2 in HCC tissues was notably higher than that in the corresponding adjacent no tumorous tissues (NT) (*n* = 112, *P* < 0.01). **b** The IHC scores of SNAIL1 in HCC tissues was notably higher than that in the corresponding adjacent no tumorous tissues (NT) (*n* = 112, *P* < 0.01). **c** In cases of high DDR2 protein expression, there was strong SNAIL1 protein expression in the same tissue section. Similarly, in the case of low DDR2 protein expression, there was no detectable SNAIL1 protein expression in the same tissue section (scale bar: 100 μm). **d** Linear regression analysis showed a significant positive correlation between ACK1 and p-ACK1 protein expression (*P* < 0.01)

Next, expression of DDR2 and SNAIL1 was assessed by immunohistochemistry in HCC and matched tumor-adjacent tissues. Both DDR2 and SNAIL1 levels were found to be evidently higher in HCC samples as compared with those in corresponding adjacent no tumorous samples (Fig. 5a-b). And there was indeed an evident positive correlation between the expression of DDR2 and SNAIL1 (*r* = 0.605, *P* < 0.01, Spearman’s correlation test) (Fig. 5c-d). To further figure out the role of DDR2 in the stabilization of SNAIL1 in HCC cells, we examined whether DDR2 directly interacted with SNAIL1 using co-immunoprecipitation assay. However, the co-immunoprecipitation measurement revealed that DDR2 protein was not bound with SNAIL1 protein directly in MHCC-97H cells (data not shown).

**Fig. 6:**
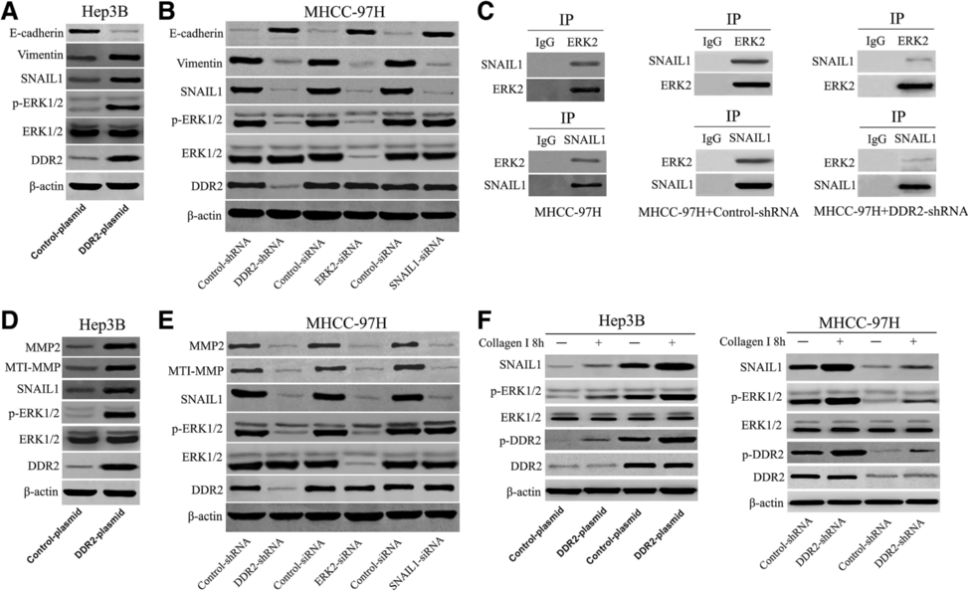
DDR2 induces EMT and up-regulates the expression of MTI-MMP and MMP2 through ERK2/SNAIL1 signaling in HCC cells. **a** Hep3B cells that had been transfected with DDR2 expressing plasmid or control plasmid, respectively, were subjected to western blotting for ERK1/2, p-ERK1/2, SNAIL1, Vimentin and E-cadherin. **b** MHCC-97H cells that had been transfected with control-shRNA, DDR2-shRNA, control-siRNA, ERK2-siRNA, control-siRNA or SNAIL1-siRNA, respectively, were subjected to western blotting for DDR2, ERK1/2, p-ERK1/2, SNAIL1, Vimentin and E-cadherin). **c** Co-immunoprecipitation assays showed that ERK2 was bound with SNAIL1 directly. **d** Hep3B cells that had been transfected with DDR2 expressing plasmid or control plasmid, respectively, were subjected to western blotting for ERK1/2, p-ERK1/2, SNAIL1, MTI-MMP and MMP2. **e** MHCC-97H cells that had been transfected with control-shRNA, DDR2-shRNA, control-siRNA, ERK2-siRNA, control-siRNA or SNAIL1-siRNA, respectively, were subjected to western blotting for DDR2, ERK1/2, p-ERK1/2, SNAIL1, MTI-MMP and MMP2. **f** HCC cells that had been transfected with indicated plasmids were added to plates coated with collagen I (2 mg/ml, BD Biosciences) for 8 h and western blotting were performed

Since ERK activation was recently shown to be critical for SNAIL1 stabilization and EMT induction, we investigated the ERK activation and its correlation with DDR2, SNAIL1 and EMT in HCC cells [29]. We found that overexpression of DDR2 in Hep3B cells increased the expression of p-ERK2, SNAIL1 and Vimentin and decreased the E-cadherin expression (Fig. 6a). DDR2 depletion in MHCC-97H cells resulted in a down-regulation of p-ERK2, SNAIL1 and Vimentin and an up-regulation of E-cadherin (Fig. 6b). ERK2 depletion in MHCC-97H cells attenuated the expression of SNAIL1, and Vimentin and increased the E-cadherin expression. (Fig. 6b). SNAIL1 depletion in MHCC-97H cells decreased the Vimentin expression and increased the E-cadherin expression (Fig. 6b). To further confirm the role of ERK2 in DDR2-induced SNAIL1 stabilization in HCC cells, we examined whether ERK2 directly interacted with SNAIL1 using co-immunoprecipitation assay. As expected, the co-immunoprecipitation measurement revealed that ERK2 protein was bound with SNAIL1 protein directly in MHCC-97H cells (Fig. 6c). Thus, these data strongly suggest that DDR2 regulates SNAIL1 stability via stimulating ERK2 activity and DDR2 may function as a key regulatory factor in HCC cell migration, invasion and EMT.

### DDR2 up-regulates MT1-MMP and MMP2 expression through ERK2/ SNAIL1 signaling in HCC

It is well known that DDR2 can regulate metalloproteinase activity in stellate cells [12]. And DDR2 expression has been associated with increased MMPs expression [13, 30]. To better understand the downstream molecules involved in DDR2 signaling-induced HCC migration and invasion, we identified whether DDR2 affected MT1-MMP and MMP2 expression in HCC cells. In our study, we found that up-regulation of DDR2 expression obviously increased MT1-MMP and MMP2 expression in Hep3B cells (Fig. 6d). In contrary, knockdown of DDR2 expression markedly decreased the MT1-MMP and MMP2 expression in MHCC-97H cells (Fig. 6e). Furthermore, we found that DDR2 up-regulated MT1-MMP and MMP2 expression through ERK2/SNAIL1 signaling in HCC cells (Fig. 6e).

### Collagen I induces DDR2/ERK2/SNAIL1 signaling activation in HCC

Considering the fact that DDR2 belongs to RTK family and type I collagen is the DDR2 ligand, we examined the effect of collagen I on the DDR2 phosphorylation. In our study, exposure of HCC cells to collagen I resulted in tyrosine phosphorylation and activation of DDR2 (Fig. 6f). Recently, it was shown that collagen I can activate DDR2/ERK2/SNAIL1 signaling axis in breast cancer. To better understand the underlying mechanism of DDR2 in HCC, we further determined whether collagen I affected DDR2/ERK2/SNAIL1 signaling activation in HCC cells. In this study, we found that collagen I significantly activates DDR2/ERK2/SNAIL1 signaling axis in HCC (Fig. 6f).

## Discussion

DDR2, a receptor tyrosine kinase, is considered to be involved in the progression of many cancer types [16, 23]. Increasing evidences have demonstrated that up-regulation of DDR2 is commonly observed in multiply tumor types including breast and prostate cancer [18, 15]. Moreover, several researches revealed that activated DDR2 could up-regulate the expression level of MMP-1, MMP-2 and MMP-13 [12, 13, 30].

Here we revealed that DDR2 expression in HCC cell lines was obviously higher than that in immortalized non-tumourigenic hepatocyte cell line L02. Our findings also showed that DDR2 expression was significantly higher in HCC tissues compared with matched normal tumor-adjacent tissues. Furthermore, high DDR2 level was evidently correlated with tumor number, vascular invasion, Edmondson-Steiner grade and TNM stage in HCC. Importantly, our results indicates that overexpression of DDR2 was obviously correlated with shorter OS and DFS, which is an assumed molecular marker for poor prognosis in HCC. Additionally, Cox repression analysis confirmed that DDR2 is a novel independent factor for HCC. Taken together, our results demonstrated that DDR2 overexpression may promote tumor progression and is a critical factor for prognosis determination in HCC patients.

In this study, we detected the phosphorylated DDR2 expression level (p-DDR2), which was previously shown to be associated with DDR2 activation. Our data indicate that DDR2 overexpression is related to the increase in activated p-DDR2. In our study, we also investigated the role of DDR2 in HCC cells. We found that up-regulation of DDR2 obviously promoted cell migration and invasion in Hep3B cells. In contrast, DDR2 depletion significantly inhibited cell migration and invasion in MHCC-97H cells. Our data suggest that DDR2 indeed facilitates the migration and invasion of HCC cell. EMT, a dynamic biological process, is crucial in the development of invasiveness and metastatic potential of many cancers including HCC [29]. The enhanced EMT process in tumor cells has been shown to increase the risk of invasion and metastasis and is associated with negative prognosis in patients with HCC [31]. Thus, we detected the expression of epithelial biomarker, E-cadherin, and mesenchymal biomarker, Vimentin with altering DDR2 expression in HCC cells. Notably, DDR2 overexpression markedly facilitates EMT and is associated with reduced E-cadherin level and elevated Vimentin level. Meanwhile, DDR2 depletion significantly inhibits EMT and is associated with elevated E-cadherin level and reduced Vimentin level. Our results revealed that DDR2 may promote HCC cell migration and invasion via inducing EMT.

Many signals generated within the cancer microenvironment activate transcription factors considered to be promoters of EMT, such as SNAIL1, TWIST1 and ZEB1 [23, 32]. Here we detected the effect of DDR2 on these transcription factors in HCC cells. Overexpression or depletion of DDR2 affected the protein expression of SNAIL1, whereas the level of TWIST1 and ZEB1 was unchanged. Notably, overexpression or depletion of DDR2 did not alter SNAIL1 mRNA expression. Furthermore, there was indeed an evident positive correlation between the expression of DDR2 and SNAIL1 in HCC tissues. Interestingly, we demonstrated that DDR2 protein was not bound with SNAIL1 protein directly in MHCC-97H cells. Our results revealed that SNAIL1 stabilization by DDR2 was unlikely to be the result of direct interaction by DDR2.

The ERK signaling is a ubiquitous signal transduction pathway that regulates important cellular processes, such as proliferation, migration and invasion [29, 33]. Increasing evidences have demonstrated that the activation of ERK is believed to contribute to tumorigenesis in many cancer types including HCC [29, 24]. Notably, ERK activation was recently shown to be critical for SNAIL1 stabilization and EMT induction [23]. Here we investigated the ERK activation and its correlation with DDR2 and SNAIL1 in HCC cells. Overexpression or depletion of DDR2 affected p-ERK2 expression, whereas the level of p-ERK1 was unchanged. Moreover, ERK2 depletion attenuated the SNAIL1 level in HCC cells. Importantly, ERK2 protein was bound with SNAIL1 protein directly in HCC cells. Our data strongly suggest that DDR2 regulates SNAIL1 stability via stimulating ERK2 activity.

MMPs activity has been related with cancer cell migration and invasion [28]. As two members of MMPs, MT1-MMP and MMP2 are critical factors in the migration and invasion of HCC [34, 28]. Considering the fact that a key biological function of RTKs is to regulate the expression of MMPs, we identified whether DDR2 affected MT1-MMP and MMP2 expression in HCC cells [12, 13]. We found that up-regulation of DDR2 expression obviously increased MT1-MMP and MMP2 expression and DDR2 depletion markedly decreased the MT1-MMP and MMP2 expression. Furthermore, DDR2 up-regulates MT1-MMP and MMP2 expression through ERK2/SNAIL1 signaling in HCC cells. These data suggest that DDR2-induced ERK2/SNAIL1 signaling activation may be responsible for the progression of EMT and up-regulation of MT1-MMP and MMP2 expression, which in turn facilitates migration and invasion of HCC.

DDR2 belongs to RTK family and type I collagen is the DDR2 ligand. Recently, it was shown that the type I collagen can activate DDR2/ERK2/SNAIL1 signaling axis in breast cancer. In our study, we found that collagen I can activate DDR2/ERK2/SNAIL1 signaling axis in HCC cells. Our results are consistent with previous findings on breast cancer, suggesting the type I collagen can cause DDR2 phosphorylation and induce DDR2/ERK2/SNAIL1 signaling axis hyperactivation.

In summary, we found that DDR2 expression level is up-regulated in HCC as compared with non-cancerous liver samples and that overexpression of DDR2 is correlated with clinicopathological characteristics of poor prognosis in HCC. Additionally, we found that DDR2 level is an independent factor for predicting the overall survival and disease-free survival of patients with HCC. Notably, DDR2 can facilitate cell invasion, migration and epithelial–mesenchymal transition via activating ERK signaling and stabilizing SNAIL1 and up-regulate MT1-MMP and MMP2 expression through ERK2/SNAIL1 signaling in HCC.

## Conclusions

Our study provides a better understanding on both the molecular mechanism and functional role of DDR2 in human HCC. Our current work revealed that DDR2 has an oncogenic role in hepatocarcinogenesis by facilitating cancer cell invasion, migration and EMT via activating ERK2 and stabilizing SNAIL1. Notably, DDR2 can up-regulate MT1-MMP and MMP2 expression through ERK2/SNAIL1 signaling in HCC. Our study identified that DDR2 is a novel regulator of EMT via DDR2-induced ERK2/SNAIL1 signaling activation, indicating its potential therapeutic value for reducing HCC invasion and metastasis.
